# The National Children’s Study: A Critical National Investment

**DOI:** 10.1289/ehp.112-1247577

**Published:** 2004-10

**Authors:** Leonardo Trasande, Philip J. Landrigan

**Affiliations:** Center for Children’s Health and the Environment, Department of Community and Preventive Medicine, New York, NY, E-mail: leo.trasande@mssm.edu; Center for Children’s Health and the Environment, Department of Community and Preventive Medicine, New York, NY, E-mail: phil.landrigan@mssm.edu

Patterns of disease in American children have changed dramatically in the past 200 years. Acquired immunodeficiency syndrome (AIDS), severe acute respiratory syndrome (SARS), and tuberculosis notwithstanding, vaccines, antibiotics, and improved hygiene have controlled the classic infectious diseases. Infant mortality has decreased by 90%. Life expectancy has nearly doubled. Yet amid this success a new challenge has arisen. Chronic diseases have increased sharply in incidence and have become the leading causes of childhood illness:

- Asthma incidence and mortality have more than doubled [Centers for Disease Control and Prevention (CDC) 1995a, 1995b].
- Despite declining mortality, incidence of acute lymphocytic leukemia increased by 61.7% from 1973 to 1999 (Robison et al. 1995).
- Incidence of primary brain cancer increased by 39.6% from 1973 to 1994 (Schechter 1999).
- Birth defects of the male reproductive system, such as hypospadias, doubled in frequency from 1970 to 1993 (Paulozzi et al. 1997).
- Neurodevelopmental disorders—including learning disabilities, dyslexia, mental retardation, attention deficit disorder, and autism—are highly prevalent and affect 5–10% of the 4 million babies born in the United States each year (Bertrand et al. 2001; CDC 2004a, 2004b; LeFever et al. 1999; Safer et al. 1996; Zito et al. 2000).
- Prevalence of childhood obesity has trebled (Galvez et al. 2003).
- Incidence rates of chronic neurodegenerative diseases of late life such as Parkinson disease and dementia and of certain cancers have increased markedly, raising the possibility of early-life antecedents (Cory-Slechta et al., unpublished data).

Although much remains to be learned about the causes of these trends, evidence is accumulating that environmental factors make important contributions.

- Airborne fine particulates have been shown to cause asthma and to trigger asthmatic atttacks (Salam et al. 2004).
- Ozone, oxides of nitrogen, environmental tobacco smoke, and indoor air pollutants all are now recognized triggers for asthma (Suh et al. 2000; Wallace et al. 2003).
- Childhood cancer has long been linked to ionizing radiation. More recently, benzene, 1,3-butadiene, and pesticides have been etiologically associated (Daniels et al. 2001; Lee et al. 2004).
- Neurobehavioral impairment has been observed following exposure of the fetal brain to even low levels of lead (Baghurst et al. 1987; Canfield et al. 2003; Dietrich et al. 1987; Lanphear et al. 2000; Opler et al. 2004; Wasserman et al. 2000), methyl mercury (Grandjean et al. 1997, 2004; Kjellstrom et al. 1986, 1989; Murata et al. 2004; National Research Council 2000), pesticides (Berkowitz et al. 2004; Perera et al. 2003), polychlorinated biphenyls (Jacobson and Jacobson 1996), and ethanol (Lupton et al. 2004). A recent National Academy of Sciences study (2000) suggests that at least 28% of developmental disabilities in children are caused by environmental factors acting alone or in concert with genetic susceptibility.

Until now, progress in elucidating the role of the environment in chronic childhood disease has been slow and incremental. Nearly all studies have examined relatively small populations of children; have considered only one chemical toxicant at a time; have had little statistical power to examine interactions among chemical, social, and behavioral factors in the environment; have had limited ability to examine gene–environment interactions (Olden 2004); and have suffered from brief duration of follow-up. Also, many previous studies have been retrospective in design and thus have been forced to estimate past exposures from limited and sometimes biased historical data.

To overcome these difficulties, the President’s Task Force on Environmental Health and Safety Risks to Children recommended in 1998 (U.S. Department of Health and Human Services 2004) that a large prospective epidemiologic study of American children be undertaken. In response, the U.S. Congress through the Children’s Health Act of 2000 authorized the National Institute of Child Health and Human Development (NICHD) “to conduct a national longitudinal study of environmental influences (including physical, chemical, biological and psychosocial) on children’s health and development” (Children’s Health Act 2000). The National Institute of Environmental Health Sciences (NIEHS), the CDC, and the U.S. Environmental Protection Agency (EPA) join the NICHD in planning and conducting this study.

Key features of this far-reaching study—now termed the National Children’s Study (NCS)—are that it will follow a representative sample of 100,000 American children from early pregnancy through age 21; a subset maybe recruited before conception. Exposure histories and biologic samples will be obtained during pregnancy and from children as they grow, obviating the need for retrospective assessments of exposures. The large sample size will facilitate simultaneous examination of the effects of multiple chemical exposures, of interactions among them, and of interactions among biologic, chemical, behavioral, and social factors. Each child will be screened genetically, thus permitting study of gene–environment interactions. Follow-up will extend over decades. For the past four years, working groups convened by NICHD have been developing the NCS: formulating core hypotheses, delineating research protocols, and planning logistics. The study is now ready for the field.

Previous major prospective epidemiologic studies have yielded invaluable gains in knowledge of disease causation and have provided critical tools for prevention and treatment. The Framingham Heart Study (Framingham, MA), for example, was established in 1948 at a time when heart disease and stroke were epidemic in the United States. The goal was to identify preventable risk factors. Within a few years, data from Framingham identified cigarette smoking (Dawber 1960) and elevated cholesterol and hypertension as preventable causes of cardiovascular disease (CVD) (Kannel et al. 1961, 1978); later analyses elucidated the role of elevated triglycerides, sedentary lifestyle, and diabetes. This information provided the blueprint for the major reductions in incidence of CVD that we have achieved in the United States over the past four decades (CDC 1999).

We anticipate that the NCS will yield equally enormous societal benefits. Six of the chronic diseases that the study plans to examine —obesity, injury, asthma, diabetes, schizophrenia, and autism—cost America $642 billion per year (Bromet and Fennig 1999; CDC 2004a, 2004b, 2004c, 2004d, 2004e, 2004f, 2004g; National Alliance for Autism Research 2002; U.S. Department of Health and Human Services 2001; Weiss 2001; Yeargin-Allsop et al. 2002). If the NCS were to produce a reduction of only 1% in incidence of these diseases, the annual savings would amount to $6.4 billion, far more than the $2.7 billion price tag of the study over 25 years.

Despite the enormous potential of the NCS, its funding is in critical jeopardy. In each of the past 4 years, the annual budget has been $12 million, a sum provided by contributions from the NICHD, the NIEHS, the CDC, and the U.S. EPA. But now to move the study forward, there is need in 2005 to establish a data-coordinating center, a repository for secure storage of biologic samples, and a series of regionally distributed vanguard recruitment sites. For these tasks, NICHD needs $15 million in new dollars above their regular budget. Without at least $27 million in federal funding in 2005, NICHD will likely be forced to cancel or at least postpone the study.

The NCS has benefited from strong and bipartisan leadership in Congress and from the support of a broad-based coalition that includes the American Academy of Pediatrics, the U.S. Conference of Catholic Bishops, the American Chemistry Council, the Learning Disabilities Association, and the March of Dimes. But still it is in dire fiscal peril.

The NCS represents an extraordinary opportunity. If the study receives the funding that it needs in 2005, it will begin as early as 2009 to produce data that will save children’s lives and improve their health. The NCS is a national investment in the future that for the sake of our children we must make today.

## Figures and Tables

**Figure:**
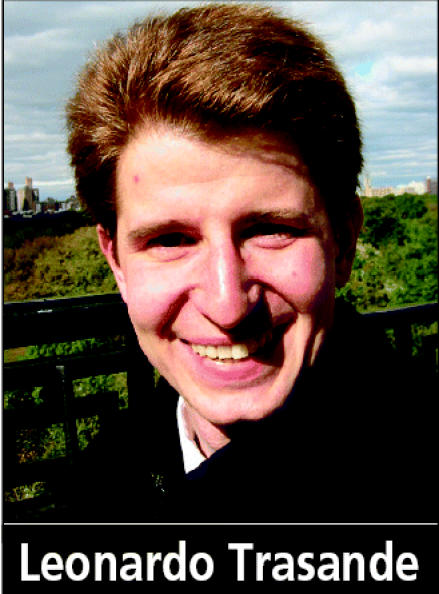


**Figure:**
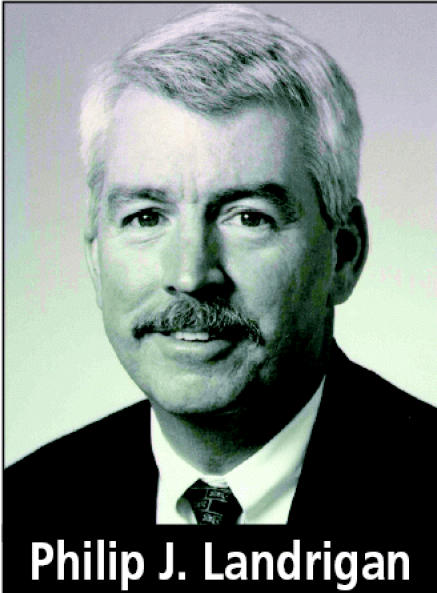


## References

[b1-ehp0112-a00789] Baghurst PA, Robertson EF, McMichael AJ, Vimpani GV, Wigg NR, Roberts RR (1987). The Port Pirie Cohort Study: lead effects on pregnancy outcome and early childhood development. Neurotoxicology.

[b2-ehp0112-a00789] Berkowitz GS, Wetmur JG, Birman-Deych E, Obel J, Lapinski, RH, Godbold JH (2004). *In utero* pesticide exposure, maternal paraoxonase activity, and head circumference. Environ Health Perspect.

[b3-ehp0112-a00789] Bertrand J, Mars A, Boyle C, Bove F, Yeargin-Allsopp M, Decoufle P (2001). Prevalence of autism in a United States population: the Brick Township, New Jersey, investigation. Pediatrics.

[b4-ehp0112-a00789] Bromet EJ, Fennig S (1999). Epidemiology and natural history of schizophrenia. Biol Psychiatry.

[b5-ehp0112-a00789] Canfield RL, Henderson CR, Cory-Slechta DA, Cox C, Jusko TA, Lanphear BP (2003). Intellectual impairment in children with blood lead concentrations below 10 microg per deciliter. N Engl J Med.

[b6-ehp0112-a00789] CDC (1995a). Children at risk from ozone air pollution, 1991–1993. MMWR Morb Mortal Wkly Rep.

[b7-ehp0112-a00789] CDC (1995b). Asthma—United States, 1982–1992. MMWR Morb Mortal Wkly Rep.

[b8-ehp0112-a00789] CDC (1999). Achievements in public health, 1900–1999: decline in deaths from heart disease and stroke—United States, 1900–1999. MMWR Morb Mortal Wkly Rep.

[b9-ehp0112-a00789] CDC 2004a. Developmental Disabilities. Available: http://www.cdc.gov/ncbddd/dd/default.htm [Accessed 21 June 2004].

[b10-ehp0112-a00789] CDC (2004b). Economic costs associated with mental retardation, cerebral palsy, hearing loss, and vision impairment—United States 2003. MMWR Morb Mortal Wkly Rep.

[b11-ehp0112-a00789] CDC 2004c. National Asthma Control Program. Available: http://www.cdc.gov/nceh/airpollu-tion/asthma/asthmaAAG.htm [Accessed 30 August 2004].

[b12-ehp0112-a00789] CDC 2004d. National Diabetes Fact Sheet: General Information and National Estimates on Diabetes in the United States. Available: http://www.cdc.gov/diabetes/pubs/factsheet.htm [Accessed 30 August 2004].

[b13-ehp0112-a00789] CDC 2004e. National Center for Injury Prevention and Control. Injuries among Children and Adolescents. Available: http://www.cdc.gov/ncipc/factsheets/children.htm [Accessed 30 August 2004].

[b14-ehp0112-a00789] CDC 2004f. Cost of Injury in the United States: A Report to Congress. Available: http://www.cdc.gov/ncipc/pub-res/cost_of_injury/ch2-3.pdf [Accessed 13 September 2004].

[b15-ehp0112-a00789] CDC 2004g. National Center for Health Statistics. Asthma Prevalence, Health Care Use and Mortality, 2000–2001. Available: http://www.cdc.gov/nchs/products/pubs/pubd/hestats/asthma/asthma.htm [Accessed 30 August 2004].

[b16-ehp0112-a00789] Children’s Health Act of 2000 2000. Public Law 106–310.

[b17-ehp0112-a00789] Daniels JL, Olshan AF, Teschke K, Hertz-Picciotto I, Savitz DA, Blatt J (2001). Residential pesticide exposure and neuroblastoma. Epidemiology.

[b18-ehp0112-a00789] Dawber TR (1960). Summary of recent literature regarding cigarette smoking and coronary heart disease. Circulation.

[b19-ehp0112-a00789] Dietrich KN, Krafft KM, Bornschein RL, Hammond PB, Berger O, Succop PA (1987). Low-level fetal lead exposure effect on neurobehavioral development in early infancy. Pediatrics.

[b20-ehp0112-a00789] Grandjean P, Murata K, Budtz-Jorgensen E, Weihe P (2004). Cardiac autonomic activity in methylmercury neurotoxicity: 14-year follow-up of a Faroese birth cohort. J Pediatr.

[b21-ehp0112-a00789] Grandjean P, Weihe P, White RF, Debes F, Araki S, Yokoyama K (1997). Cognitive deficit in 7-year-old children with prenatal exposure to methylmercury. Neurotoxicol Teratol.

[b22-ehp0112-a00789] Galvez MP, Frieden TR, Landrigan PJ (2003). Obesity in the 21st century. Environ Health Perspect.

[b23-ehp0112-a00789] Jacobson JL, Jacobson SW (1996). Intellectual impairment in children exposed to polychlorinated biphenyls *in utero*. N Engl J Med.

[b24-ehp0112-a00789] Kannel WB, Dawber TR, Kagan A, Revotskie N, Stokes JI (1961). Factors of risk in the development of coronary heart disease—six year follow-up experience; the Framingham Study. Ann Intern Med.

[b25-ehp0112-a00789] Kannel WB, Wolf PA, Dawber TR (1978). Hypertension and cardiac impairments increase stroke risk. Geriatrics.

[b26-ehp0112-a00789] Kjellstrom T Kennedy P Wallis S Mantell C 1986. Physical and Mental Development of Children With Prenatal Exposure to Mercury From Fish. Stage I: Preliminary Tests at Age 4. Report 3080. Solna, Sweden:National Swedish Environmental Protection Board.

[b27-ehp0112-a00789] Kjellstrom T Kennedy P Wallis S Stewart A Friberg L Lind B 1989. Physical and Mental Development of Children With Prenatal Exposure to Mercury From Fish. Stage II: Interviews and Psychological Tests at Age 6. Report 3642. Solna, Sweden:National Swedish Environmental Protection Board.

[b28-ehp0112-a00789] Lanphear BP, Dietrich K, Auinger P, Cox C (2000). Cognitive deficits associated with blood lead concentrations < 10 microg/dL in US children and adolescents. Public Health Rep.

[b29-ehp0112-a00789] Lee WJ, Cantor KP, Berzofsky JA, Zahm SH, Blair A (2004). Non-Hodgkin’s lymphoma among asthmatics exposed to pesticides. Int J Cancer.

[b30-ehp0112-a00789] LeFever GB, Dawson KV, Morrow AL (1999). The extent of drug therapy for attention deficit-hyperactivity disorder among children in public schools. Am J Public Health.

[b31-ehp0112-a00789] Lupton C, Burd L, Harwood R (2004). Cost of fetal alcohol spectrum disorders. Am J Med Genet.

[b32-ehp0112-a00789] Murata K, Weihe P, Budtz-Jorgensen E, Jorgensen PJ, Grandjean P (2004). Delayed brainstem auditory evoked potential latencies in 14-year-old children exposed to methylmercury. J Pediatr.

[b33-ehp0112-a00789] National Academy of Sciences, Committee on Developmental Toxicology 2000. Scientific Frontiers in Developmental Toxicology and Risk Assessment. Washington, DC:National Academies Press. 25077274

[b34-ehp0112-a00789] National Alliance for Autism Research 2002. What is Autism? Available: http://www.naar.org/aboutaut/whatis.htm [Accessed 30 August 2004].

[b35-ehp0112-a00789] National Research Council 2000. Toxicological Effects of Methylmercury. Washington, DC: National Academies Press. 25077280

[b36-ehp0112-a00789] Olden K (2004). Genomics in environmental health research—opportunities and challenges. Toxicology.

[b37-ehp0112-a00789] Opler MG, Brown AS, Graziano J, Desai M, Zheng W, Schaefer C (2004). Prenatal lead exposure, δ-aminolevulinic acid, and schizophrenia. Environ Health Perspect.

[b38-ehp0112-a00789] Paulozzi LJ, Erickson JD, Jackson RJ (1997). Hypospadias trends in two US surveillance systems. Pediatrics.

[b39-ehp0112-a00789] Perera FP, Rauh V, Tsai WY, Kinney P, Camann D, Barr D (2003). Effects of transplacental exposure to environmental pollutants on birth outcomes in a multiethnic population. Environ Health Perspect.

[b40-ehp0112-a00789] Robison LL, Buckley JD, Bunin G (1995). Assessment of environmental and genetic factors in the etiology of childhood cancers: the Childrens Cancer Group epidemiology program. Environ Health Perspect.

[b41-ehp0112-a00789] Safer DJ, Zito JM, Fine EM (1996). Increased methylphenidate usage for attention deficit disorder in the 1990s. Pediatrics.

[b42-ehp0112-a00789] Salam MT, Li YF, Langholz B, Gilliland FD, Children’s Health Study (2004). Early-life environmental risk factors for asthma: findings from the Children’s Health Study. Lancet.

[b43-ehp0112-a00789] Schechter CB (1999). Re: Brain and other central nervous system cancers: recent trends in incidence and mortality. J Natl Cancer Inst.

[b44-ehp0112-a00789] Suh HH, Bahadori T, Vallarino J, Spengler JD (2000). Criteria air pollutants and toxic air pollutants. Environ Health Perspect.

[b45-ehp0112-a00789] U.S. Department of Health and Human Services The President's Task Force on Environmental Health Risks and Safety Risks to Children. Available: http://nationalchildrensstudy.gov/about/task_force.cfm [Accessed 13 September 2004].

[b46-ehp0112-a00789] U.S. Department of Health and Human Services Public Health Service, Office of the Surgeon General. 2001. The Surgeon General’s Call to Action to Prevent and Decrease Overweight and Obesity. Available: http://www.surgeongeneral.gov/topics/obesity/calltoaction/CalltoAction.pdf [Accessed 30 August 2004]. 20669513

[b47-ehp0112-a00789] Wallace LA, Mitchell H, O’Connor GT, Neas L, Lippmann M, Kattan M (2003). Particle concentrations in inner-city homes of children with asthma: the effect of smoking, cooking, and outdoor pollution. Environ Health Perspect.

[b48-ehp0112-a00789] Wasserman GA, Liu X, Popovac D, Factor-Litvak P, Kline J, Waternaux C (2000). The Yugoslavia Prospective Lead Study: contributions of prenatal and postnatal lead exposure to early intelligence. Neurotoxicol Teratol.

[b49-ehp0112-a00789] Weiss KB, Sullivan SD (2001). The health economics of asthma and rhinitis. I. Assessing the economic impact. J Allergy Clin Immunol.

[b50-ehp0112-a00789] Yeargin-Allsopp M, Rice C, Karapurkar T, Doernberg N, Boyle C, Schendel D (2002). Prevalance of autism in a US metropolitan city. JAMA.

[b51-ehp0112-a00789] Zito JM, Safer DJ, dosReis S, Gardner JF, Boles M, Lynch F (2000). Trends in the prescribing of psychotropic medications to preschoolers. JAMA.

